# The skill of self-control

**DOI:** 10.1007/s11229-021-03068-w

**Published:** 2021-02-18

**Authors:** Juan Pablo Bermúdez

**Affiliations:** 1 Institut de Philosophie, Université de Neuchâtel, Neuchâtel, Switzerland; 2 Programa de Filosofía & Área de Investigación Salud, Conocimiento Médico y Sociedad, Universidad Externado de Colombia, Bogotá, Colombia

**Keywords:** Cognitive control, Guidance, Self-regulation, Strategies, Effort, Dual-process, Metacognition, Reasons, Agency

## Abstract

Researchers often claim that self-control is a skill. It is also often stated that self-control exertions are intentional actions. However, no account has yet been proposed of the skillful agency that makes self-control exertion possible, so our understanding of self-control remains incomplete. Here I propose the *skill model of self-control*, which accounts for skillful agency by tackling the guidance problem: how can agents transform their abstract and coarse-grained intentions into the highly context-sensitive, fine-grained control processes required to select, revise and correct strategies during self-control exertion? The skill model borrows conceptual tools from ‘hierarchical models’ recently developed in the context of motor skills, and asserts that self-control crucially involves the ability to manage the implementation and monitoring of regulatory strategies as the self-control exercise unfolds. Skilled agents are able do this by means of *flexible practical reasoning*: a fast, context-sensitive type of deliberation that incorporates non-propositional representations (including feedback signals about strategy implementation, such as the feeling of mental effort) into the formation and revision of the mixed-format intentions that structure self-control exertion. The literatures on implementation intentions and motivation framing offer corroborating evidence for the theory. As a surprising result, the skill of self-control that allows agents to overcome the contrary motivations they experience is self-effacing: instead of continuously honing this skill, expert agents replace it with a different one, which minimizes or prevents contrary motivations from arising in the first place. Thus, the more expert you are at self-control, the less likely you are to use it.

Tom and his partner have recently committed to saving all the money they can to buy a house. While doing groceries, Tom stumbles upon the wine section. His eyes drift toward a rare Burgundy bottle, and he discovers that it’s on sale today. Noticing a growing desire to buy the bottle, to protect his commitment to save from being overpowered, he tries to imagine the feeling of opening the door to his new home. But this Pinot Noir’s immediate allure is too strong, and the motivational conflict inside Tom intensifies. So he tries to distract himself from the bottle by turning away from it and looking at the unchecked items in his shopping list. But he finds himself surrounded by wine, which does not help. In a last attempt, Tom calls his husband Tim and tells him about his dilemma. Tim sternly reminds Tom they have enough wine at home, so he should leave that and go back to his chore at once. That does the trick for him.

This story illustrates a fact about self-control often unacknowledged in philosophical discussion: agents can actively select, revise, and correct the self-control strategies they deploy while self-control exertion unfolds. Dozens of possible strategies exist to tackle motivational conflicts, from selecting which situations we enter and restructuring our environments, to reconceptualizing stimuli and forcing ourselves to inhibit a behavioral tendency (Naragon-Gainey et al. 2017); and evidence shows people often use multiple strategies in a single self-control episode (Aldao and Nolen-Hoeksema 2013; Ford et al. 2019). That we try actively to select and manage self-control strategies is also revealed by the flowing stream of popular books offering tips and tricks to improve willpower, overcome procrastination, and break bad habits.^1^ Additionally, programs and groups tackling addiction are a staple of psychological research and practice; and countless online services and apps promise to help you manage attention, generate and sustain good habits, and advance along the path of self-improvement.

Selecting and managing self-control strategies is a tricky endeavor. While some strategies may be generally more effective than others (Aldao et al. 2010; Duckworth et al. 2016), a strategy’s success depends significantly on the particular context and the agent’s abilities (Bonanno and Burton 2013), so appropriately managing self-control strategies requires a great deal of skill. Agents must be able to determine which among multiple strategies best fits the current context; whether the strategy implementation is working or not; and either maintain or revise the strategy as needed. It thus seems right to recognize, as theorists often have, that successful self-control exertion requires skill (Levy 2017; Mele 1987), and that self-control is itself a complex skill (Holton 2009; Metcalfe and Mischel 1999).

And yet, no complete account has been proposed of the skillful agency that makes self-control exertion possible.^2^ Such an account must solve the *guidance problem*: how can agents transform their abstract and coarse-grained commitments into the highly context-sensitive, fine-grained control processes required to select, revise and correct strategies during self-control exertion? As I will show, existing explanations identify the agent’s involvement with deliberation and commitment formation processes that occur prior to self-control exertion (e.g. Tom’s deciding he will save to buy a house), but do not yet account for the crucial jobs the agent must perform while exerting self-control: selecting an appropriate strategy, specifying its implementation parameters, monitoring its success, and maintaining or modifying it as appropriate. Current theories have not said enough to explain how agents can control self-control exertion as it unfolds.

Here I develop the *skill model of self-control* employing recent conceptual developments from the literature on motor skills. Section 1 presents the guidance problem and explains why it has not yet been solved. Section 2 introduces hierarchical models of skillful motor agency, and Sect. 3 builds a skill model that presents self-control exertion as a skillful action and is capable of solving the guidance problem. Section 4 unpacks some of the skill model’s implications for the philosophy of self-control.

This discussion focuses on *synchronic* self-control: the ability to overcome a temptation that is currently active and motivationally stronger than the commitment it opposes.^3^ This is distinct from other possible forms of self-control, particularly *diachronic* self-control: the ability to overcome foreseeably, but not currently, dominant temptations. Accordingly, ‘self-control’ will henceforth refer to synchronic self-control unless otherwise noted. That said, one of this account’s upshots is clarifying the relationship between synchronic and diachronic self-control. Specifically, I will argue that synchronic self-control has explanatory primacy, but diachronic self-control has practical prevalence: the former makes the latter’s development possible, but the latter is used more frequently by skilled agents. This leads to the surprising conclusion that synchronic self-control is self-effacing: the more skilled at it you are, the less you tend to use it.

## The guidance problem

### The skillful agency requirement

The view that self-control exertions are something we intentionally do, not merely something that happens to us, is widely shared (Holton 2009; Mele 1998; Sripada 2014; but see Kennett and Smith 1996). Everyday responsibility attribution practices also presuppose this: if we are to consider people praiseworthy or blameworthy for exerting self-control or failing to do so, we must assume that exercising self-control is in some significant sense under their control and up to them.^4^ Intuitively, self-control is something we intentionally do: a process we exert to transform our commitments into action despite the motivational obstacles we find along the way, and to protect our coherence as planning agents acting through time.

A consensus is also growing among philosophers and scientists that rather than a resource (like energy or glucose) or a capacity (like working memory), self-control is rather a *skill* (Bermúdez 2021; Inzlicht et al. 2020; Levy 2017; Mischel 2014; Moffitt et al. 2011; Tabibnia et al. 2011). Mele holds self-control is not “a mental analog of brute physical strength” since, unlike resources or mere capacities, its acquisition requires a gradual learning process that leads to developing “a variety of skills—and considerable savvy about which skills to use in particular situations” (2011, pp. 468–469). Additionally, self-control exertion often spans “across multiple psychological domains, i.e., action selection, attention, belief, evaluation, memory, and thought” and “requires performing the right cognitive control actions at the right time with the right intensity for the right duration” (Sripada 2020). Thus guiding self-control exertion is not simply a matter psychological capacity, but rather a matter of coordinating capacities to solve a self-control problem. Such complex, multi-track ability belongs in the category of skills, rather than that of uni-dimensional resources or capacities.

I take it as a starting point, then, that a theory of self-control must satisfy the *skillful agency requirement*: it must explain how self-control exertions can be intentional actions in the particular ways in which skillful actions are.^5^ This is challenging because it requires solving the guidance problem.

### Guidance

Self-control is required when an agent undergoes a motivational conflict, i.e. a conflict between a *commitment* (e.g. an intention or a judgment about what is all-things-considered best to do) and a *temptation*, i.e. a commitment-discordant motivational state (this could be a desire, a craving, an emotion, or an urge).^6^ Motivational conflicts usually admit of a broad range of solutions. Consider Walter Mischel’s work on delay of gratification, where four- and five-year-old children were placed in front of a yummy treat (often a marshmallow) and told they could either eat the one treat or wait until the experimenter returned (usually 10–15 min) and get two treats instead. Successful children used multiple distinct strategies:

some put their hands over their eyes, rested their heads on their arms, and invented other similar techniques for averting their gaze most of the time, occasionally seeming to remind themselves with a quick glance. Some talked quietly to themselves or even sang (‘This is such a pretty day, hooray’); others made faces, picked their noses, made up games with their hands and feet, and even tried to doze off while continuing to wait. One of the most successful ‘delayers’ actually managed to nap during the delay time (Mischel 1996, p. 202).

Different strategies had strikingly different results (Mischel et al. 1989). If instructed to think “fun thoughts”, children waited twice as long as those told to focus on the reward they would get. Children told to focus on how the marshmallow looked like a fluffy cloud waited on average almost three times more than those told to focus on the marshmallow’s chewy sweetness (13 min vs. less than 5 min). And those told to imagine the marshmallow as a *picture* of a marshmallow waited for an average 17 min. The striking influence of verbal instructions on performance shows that intentional strategy selection affects self-control effectiveness. This illustrates that there are several crucial jobs for the agent to do as the self-control episode unfolds: specify how the commitment is to be implemented, monitor the implementation’s success, and solve problems that arise during implementation. In a word, after the commitment is formed and self-control exertion is initiated, the agent needs to *guide* self-control exertion as it unfolds.

How is such guidance to be explained? So far, attempts to tackle this question have appealed to a series of information-accumulation mechanisms used in cognitive science to explain decision-making. Consider Chandra Sripada’s (2020) ‘divided mind’^7^ theory, which holds that self-control exertion is constituted by a series of skillfully orchestrated cognitive control processes aimed at overcoming commitment-discordant automatic response tendencies. *Cognitive control* is the set of higher-order cognitive functions dedicated to producing goal-directed behavior, particularly when this requires overcoming competing response tendencies. It has three key functions (Shenhav et al. 2013). First, *monitoring for conflict signals* that occur when the agent is performing some goal-directed behavior (e.g. performance delays or a mismatch between goal-state representation and perceptual outcome). Second, *selecting a control strategy*, i.e. selecting which regulatory processes must be implemented to solve the conflict. Third, *implementing the control strategy* through regulatory mechanisms like inhibitory control and working memory. Applying this to self-control, Sripada argues that agents solve motivational conflicts by deploying their cognitive control resources: if sufficiently salient, the conflict between temptation and commitment is detected by cognitive control’s monitoring systems; cognitive control then selects a control strategy to overcome the temptation in favor of the agent’s commitment, and recruits regulatory resources to implement the selected strategy.^8^

To account for guidance, Sripada refers to cognitive control’s selection mechanism. This mechanism subpersonally performs cost–benefit calculations to estimate the “expected value of control” [EVC] for each available strategy and selects the expected-value-maximizing one. Sripada argues that this and other such processes generate exercises of self-control by forming “executive decisions”: subpersonal processes that integrate and accumulate information in favor of selecting a control strategy until it reaches a critical threshold, at which point a decision is made whether to implement the strategy or not. These are, of course, not *decisions* in the traditional sense—they involve no conscious weighting of reasons. Such ‘bounded accumulation’ or ‘sequential sampling’ models (Forstmann et al. 2016), used to model fast decision-making processes, portray processes that occur subpersonally, just like EVC calculations. Even if the decision itself can become accessible at the personal level once it is produced, the decision-making (i.e. the information-accumulation and assessment) processes remain subpersonal.^9^

While these information-aggregation mechanisms must certainly play a crucial role (see Sect. 3.1 below), crucial elements of the story are still missing. If decision-making occurs largely at the subpersonal level (the level of neural sub-systems independent of the agent’s experience), this raises the concern that, by appealing to these processes to account for agency, we lose sight of what we wanted to explain. After all, as Adina Roskies (2018) argues,

if decision-making is merely a competition between incoming sensory information in favor of one of two options, decisions can be reached without control, without awareness, and perhaps even without the operation of mind entirely. Self-governance seems out of the question: There is no “self” to do the self-governing, or to establish self-control (p. 248).

In response it can be argued that these information-accumulation processes are agential in an important deliberative sense. After all, they are triggered by a conflict between a motivational state and the agent’s commitment, and their goal is identifying the value-maximizing strategy to protect the agent’s commitment. Thus, insofar as they are sensitive to the agent’s commitment, they are sensitive to the process of practical reasoning that originated it. Arguably, then, these processes are agentive, even if subpersonal, since they are reasons-responsive.

Even granting this, the story so far remains crucially incomplete. A theory of agency needs to explain not only the action’s relationship to the agent’s reasons, but also the agent’s relationship to her action as it unfolds (Frankfurt 1978). It should explain not only how actions are reasons-responsive, but also how agents are able to shape them as they occur. And this requires a story of personal-level guidance. Lacking this, agency in self-control would be analogous to driving a fully autonomous car: we tell it where we want to go, we press the button, and the car runs its mechanisms to yield a result that matches our goal; but we are not in control of its speed, the force with which it stops, or how it handles pedestrian interactions; we exert no control over whether the car’s mechanisms succeed or fail, despite having played an agentive role in triggering them and setting their target.

Thus, with respect to self-control the *guidance problem* remains unsolved: how can agents structure and manage the cognitive processes that constitute self-control exertions as these processes unfold? Successfully solving this problem will require not only providing a personal-level story of agentive control during self-control exertion, but also explaining how the personal-level processes that feature in this story interact with subpersonal processes of the kind that compose information-aggregation mechanisms like EVC and others.

Fortunately, we have a clue about where to look for solutions to the guidance problem: if self-control is a skill, the guidance account should be similar to that used to explain other skillful actions. Motor skill researchers have recently been hard at work tackling the issue of how personal and subpersonal processes interact in action control to enable agentive guidance. The next section will introduce tools developed to explain guidance of motor action, which Sects. 3–4 will apply to self-control.

## Skillful guidance in motor action

The study of skillful motor action involves the challenge of explaining how agentive performance can be structured by both the agent’s intentions (which specify the outcome to be achieved in a propositional format apt for deliberation and planning) and motor representations (which represent action-relevant attributes of objects and situations in a format that is constrained by the biomechanical features of the agent’s body, suitable for guiding action execution). This is challenging since intentions and motor representations have different representational formats: the former abstract and propositional, the latter fine-grained and non-propositional. The challenge is explaining how these two representation types interact in order to jointly and non-accidentally produce coherent goal-directed behavior.^10^ Attempts to face this challenge have relied on a well-confirmed ‘hierarchical framework’ of the psychological architecture of agentive control. In this section I briefly sketch a specific version of the hierarchical framework, which makes it possible to identify different levels of control and interactions between them.^11^

According to the hierarchical framework, action control works via a series of coordinated, hierarchically-arranged processing levels. Higher-level control processes manipulate more abstract, coarser-grained representations, and lower-level processes manipulate more specific, finer-grained representations. Higher-level processes influence and coordinate multiple lower-level processes, while lower-level processes somewhat autonomously ‘fill in the details’ of implementation left unspecified by higher-order ones (Pacherie 2008). (We will see, however, that influence also flows from the bottom up.)

According to ‘mixed-formats’ versions of the hierarchical model (Fridland 2019; Shepherd 2017, 2018), some intentions can carry contents with more than one format. Such mixed-format intentions connect higher-level, purely propositional intentions and lower-level, purely motor representations.^12^ At the higher level, *general intentions* propositionally represent the agent’s global plans and goals, while intermediate-level *mixed-format intentions* specify the steps and strategies selected to achieve the goal represented in the general intention, in a format capable of influencing lower-level sensory and motor processes. Performance can involve multiple layers of general and mixed-format intentions, each one refining the representational grain of the one above, further specifying the action’s implementation details.^13^

Processes of practical reasoning which are the traditional focus of philosophers are too slow for skilled action time frames, and manipulate mainly propositionally formatted representations which are too abstract and rough-grained to productively interact with motor representations. Thus, while they can adequately produce general intentions, they are not suitable to forming mixed-format intentions. These require *flexible practical reasoning*, a type of deliberation that can integrate both propositionally- and nonpropositionally-formatted representations (e.g. sensory, motor, imagistic, affective…) employing non-deductive processes (like association or imagistic rehearsal) that aim at the formation of a mixed-format intention. Flexible practical reasoning takes an already-formed general intention as its starting point, and aims at forming a mixed-format intention that further specifies the means by which the general intention can be implemented.^14^ Both propositional and flexible practical reasoning processes count as *practical reasoning* in the sense that they are conscious, deliberative processes whereby the agent aims at reaching a conclusion about what to do.

Consider the task of deciding where in an empty room a sofa should be placed. Instead of measuring the dimensions of the sofa, the room and the other objects, and then calculating an adequate location, as you look at the room you can overlay a mental image of the sofa onto different spots and select the one that seems to work best. We often use such blends of mental imagery and perception (what Briscoe (2018) calls “make-perceive”) in everyday practical reasoning (Shepherd 2018). Through these flexible (i.e. quick, context-sensitive and reasons-responsive) deliberative processes, agents can form and manage mixed-format intentions more efficiently than via traditional deliberation, while the process still respects rationality constraints like consistency with beliefs and desires, and means-end coherence. In the case of skill, flexible practical reasoning is bolstered by well-practiced perceptual, attentional, and conceptual routines that enable skilled agents to intuitively grasp relevant aspects of the situation and assess different opportunities for action.

**Fig. 1:**
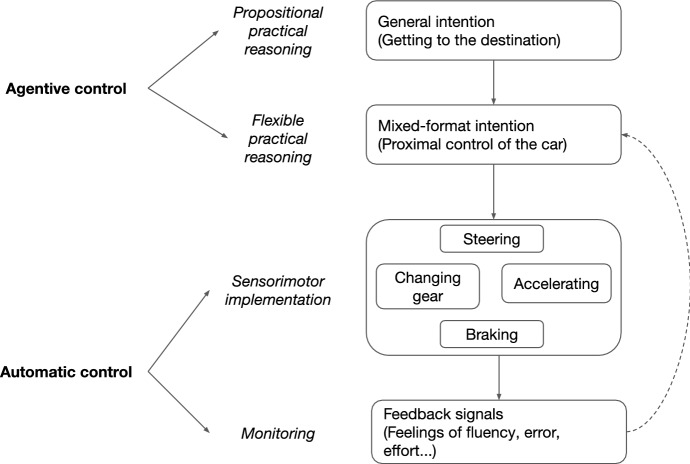
Hierarchical control structures in skilled bodily action (based on Christensen et al. 2016)

To illustrate, take the skill of driving.^15^ When a skilled driver S navigates through moving traffic, pedestrians and climate conditions to reach her destination, we can distinguish four different levels of control at play (Fig. 1).^16^ At the most abstract level, S has a general intention representing her goal (e.g. arriving at a given destination), often formed via *propositional practical reasoning*. This general intention then informs her lower-level *flexible practical reasoning*, whereby S uses variously-formatted representations (propositional, sensory, imagistic, perceptual, etc.) to select appropriate context-sensitive ways to reach her goal. S thereby selects proximal control strategies (accelerating to reach highway traffic speed; shifting lanes towards the exit; etc.) whose mixed-format representation constitutes the mixed-format intention’s content. Both types of practical reasoning are forms of agentive control: reasons-responsive, personal-level processes whereby the agent intentionally structures her behavior in light of her reasons for action.

Further down the hierarchy, *automatic control* processes implement the multiple sensorimotor routines that constitute skillful performance, while monitoring and adjusting performance as it unfolds. Implementation processes deploy fine-grained procedures that have been sufficiently trained to incorporate the reasons-responsiveness acquired through deliberate practice, and sufficiently over-practiced to become largely automatic (e.g. steering, accelerating, changing gears, using the breaks, and so on). Monitoring processes transmit error signals in the implementation process (e.g. mismatches between expected outcome and sensory feedback, or performance delays).

The frontier between agentive and automatic control shifts as agents gain experience, with more expert agents having richer automatic routines and exerting practical reasoning at higher levels. Additionally, a process that is usually automatic can become deliberate, e.g. if S decides to deliberately practice in order to improve technique. Thus, not all instances of driving involve agentive control to the same degree. Easy drives rely mostly on well-practiced, automatic routines, though arguably some minimal degree of agentive control is still involved, e.g. to remain vigilant of unforeseen obstacles. But harder drives (e.g. driving in a new city through heavy rain) will require engaging in much more constant and complex flexible practical deliberation.

Crucially, influence also flows from the bottom up. Subpersonal monitoring systems, trained through repeated practice, continually assess how the implementation is going and output error signals. Many of these signals are used by automatic control to adjust implementation parameters autonomously, but some of them also manifest in S’s experience as e.g. feelings of fluidity, surprise, error or effort. Call these *action-oriented feelings*: conscious, affect-involving representations produced by subpersonal information-accumulation processes, which provide the agent with information about how implementation is going, in non-propositional formats that she can quickly incorporate into flexible practical reasoning at the personal level. By allowing agents to make quick, informed decisions about whether to maintain or modify mixed-format intentions mid-performance (e.g. the feeling that the steering wheel is not responding as expected might lead the driver to stop and check for mechanical problems), action-oriented feelings and flexible practical reasoning are part of the psychological machinery that makes it possible for agents to guide action as it unfolds. Thus, the output of subpersonal monitoring processes, in the form of action-oriented feelings, can itself become input for new rounds of personal-level practical reasoning which adjust or revise the mixed-format intentions at play, in a feedback loop that steers performance as it happens.

Before moving on, it is important to tackle one question. I mentioned that both propositional and flexible practical reasoning are reasons-responsive; but how is the latter reasons-responsive? This is a particularly challenging issue given that the multiplicity of representational formats involved requires some sort of unification.^17^ A full response is beyond the scope of this paper, but let me quickly sketch three elements toward an answer.

I begin by suggesting that, for any sufficiently complex action *A*, forming a general intention to perform it raises for the agent an implementation question that can be formulated as *‘How will I A?’.* It need not be explicitly or linguistically articulated, but finding an answer to this question is necessary for implementing the action, and the question itself can be seen as establishing a domain of congruent answers, i.e. specifications of possible steps I can take to try to reach the goal represented in my intention. Flexible practical reasoning is just the cognitive process of attentively seeking a congruent answer to the implementation question.^18^ In seeking answers to this question, the agent’s attentional focus shifts between multiple representations (perceptual, motor, mnemonic, imagistic…) as she looks for contributions to building an appropriate answer. Through flexible practical reasoning, the agent threads several of these diverse representations together to form a congruent answer to the question posed by the agent’s general intention. This attentive search on the part of the agent explains how diverse representations can be unified to constitute the content of mixed-format intentions, which specify congruent answers to the implementation question. The more skilled the agent is, the more accurately she will be able to distinguish congruent from incongruent answers, relevant from irrelevant representations. This sets constraints on which representations are selected and which mixed-format intentions are formed as a result.

This attentive search is one component of the explanation of of flexible practical reasoning’s reasons-responsiveness. A second component is the representations and metacognitive signals that skilled agents have developed through their history of deliberate practice. Through practice, agents form what Pacherie and Mylopoulos (2020) call “structured action representations”, which bring together nodes of chunked motor schemas (mappings of sensory inputs and parameter values onto motor outcomes) in a way that is responsive to the action domain’s structure. Since they crystallize the structural features of the action domain, skilled agents can use such representations to efficiently tackle familiar situations. Representations like these make flexible practical reasoning sensitive to reasons without the need to explicitly represent such reasons in a propositional format at the time of action. By including structured action representations as part of their content, mixed-format representations can inform lower-level perceptual and motor routines in accordance with the action domain’s constraints.

Finally, when multiple possible answers to the same implementation question emerge, flexible practical reasoning is also in charge of selecting one among them which seems most promising for the current action situation. To do this, the skilled agent can rely on action-oriented feelings (e.g. feelings of fluency, of risk, and of effort) to compare the candidate answers and select the one that seems most promising. As mentioned above, the skilled agent’s practice history fine-tunes these metacognitive processes, making them both sensitive to the expected costs and benefits of each available strategy, and capable of signaling when a change of strategy is needed mid-performance. This aspect of the process further accounts for its reasons-responsiveness, and will become crucial to the discussion of self-control skill below.

I leave further questions about the hierarchical framework and flexible practical reasoning for another occasion. My goal here is showing that we can illuminate agentive guidance in self-control through the hierarchical framework.

## The skill of self-control

To build a hierarchical model of self-control guidance, I begin from the bottom up, by characterizing (3.1) the feeling of mental effort as an action-oriented feeling. I then explain how (3.2) skilled agents can use flexible practical reasoning to identify and generate effective regulatory strategies during a given motivational-conflict situation. If this is correct, a crucial prediction is that the way agents form mixed-format intentions has an influence on how successful they are at exerting self-control. I present (3.3) evidence that corroborates this prediction.

### The feeling of mental effort

Recall that part of what makes the guidance problem pressing for self-control is the wide range of self-control strategies available to agents: how do agents skillfully manage strategy selection and implementation? To answer, theorists have appealed to cognitive control’s selection function, positing a reinforcement-learning system that seeks to maximize long-term reward by identifying the control strategy that the agent’s past type-similar experiences reveal as having the highest expected value. This ‘strategy selector’ estimates the expected value of control [EVC] of a given strategy by pitting its expected benefits against its expected costs. A strategy’s expected benefits are intuitively easy to grasp. In the marshmallow case, the expected benefit of control exertion is obtaining a double reward, weighted by the probability of actually succeeding (as determined by the agent’s past experiences). To specify the costs of control, these models assume that cognitive control exertion is inherently costly. This is supported by evidence that people are willing to forego economic reward to avoid cognitively effortful tasks. From this kind of aversion to control exertion researchers infer a cost of control (Kool et al. 2010). Accordingly, the more mental effort a given strategy requires, and the longer it must be implemented, the costlier it is estimated to be. Strategy selectors calculate EVC for all available control strategies, and select the EVC-maximizing strategy. As strategy implementation carries on, selectors continue to update EVC calculations according to feedback from monitoring systems. Thus EVC-maximizing strategies can change mid-performance.

So far this description is entirely subpersonal, but the process is not meant to be. The *feeling of mental effort* [*FME*] is posited as “the conscious, experienced measurement of the costs” of control strategy implementation (Kurzban et al. 2013, p. 662); this is a personal-level representation in that it need not but may become conscious once costs are large enough (Westbrook and Braver 2015, p. 399). As an index of the costs of a given control strategy, an intensification of FME motivates the agent to search for alternatives to said strategy (which can be selecting a different strategy or no strategy at all). FME is *predictive* in the sense that it informs about the future costs of deploying a strategy. Hence FME can be used to assess strategies both before and during implementation.

FME is an action-oriented feeling. As a product of the reinforcement-learning systems that estimate control’s costs, it integrates information about the agent’s current cognitive task, her capacities and limits, and the control strategy’s expected costs in the current context. Thus FME gives the agent otherwise inaccessible action-relevant information about strategies and their expected costs. While it is a product of subpersonal, reinforcement-learning-based calculations, agents can use FME as input for flexible practical reasoning, to decide whether to maintain the current mixed-format intention or modify it at the personal level.

If this is correct, FME is well-suited for skilled action guidance in both its format—because it presents this information as an affective experience that agents can quickly incorporate into flexible practical reasoning—and its content—because it represents action-relevant information about agent, context, and strategy implementation. FME thus illustrates how to solve the guidance problem: skilled agents can guide self-control exertion by selecting, maintaining and revising control strategies through a flexible practical reasoning that is possible, among other things, by input from action-oriented feelings like FME which offer crucial information about strategy implementation.

### Skillful guidance during self-control exertion

**Fig. 2:**
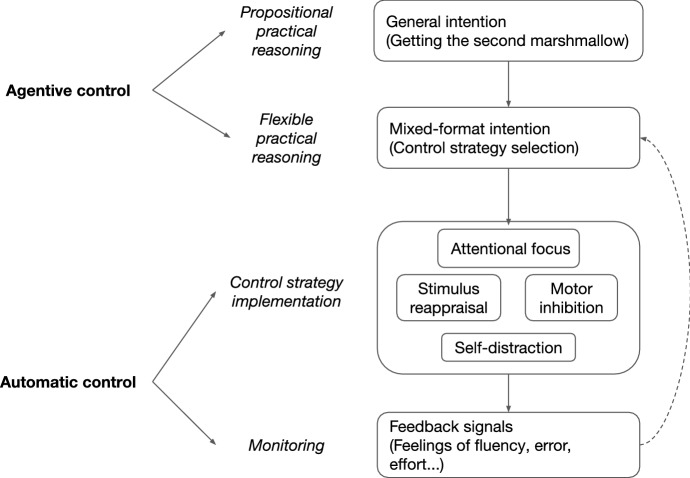
Hierarchical control structures in self-control exertions

Consider the marshmallow study children again. In navigating a motivational situation crowded with temptations, they display four levels of control (Fig. 2). At the most abstract level, the children represent the goal they have committed to: a general intention (to obtain the second marshmallow) perhaps formed via standard *propositional practical reasoning* after understanding the experimenter’s instructions. Faced with the challenge of waiting and the growing desire to immediately eat the one marshmallow, each child carries out a lower-level instance of *flexible practical reasoning*, i.e. a quick search for the right actionable means to achieve her goal in this context. This kind of reasoning takes as input action-oriented non-propositional states like mental-imagery/perception blends (e.g. imagining places in the room to hide the marshmallow) and affective representations like FME.

Skilled self-controlled agents thus use flexible practical reasoning to form, maintain and adapt *mixed-format intentions*, which represent the selected control strategy and its implementation parameters in specific ways that can be carried out by lower-level implementation and monitoring processes. These lower-level processes can include directing attention (e.g. focusing on the goal or distracting oneself), reappraising the tempting stimuli (e.g. representing the marshmallow as white and cloud-like) or inhibiting certain behavior (e.g. refraining from extending one’s hands toward the marshmallow). While implementation and monitoring are subpersonal processes, they are structured by the mixed-format intention’s content. Conversely, these subpersonal processes generate action-oriented representations like FME that agents can use to guide strategy implementation as it unfolds via flexible practical reasoning. Thus agents can use all levels of control to intentionally influence self-control exertion by forming and managing their mixed-format intention.

Now, just like easier and harder instances of driving require different amounts of agentive control, similarly with easier and harder instances of self-control exertion. Synchronic self-control always implies some difficulty: the automatic tendency is to follow temptation, so cognitive control must be recruited to counter it. But just like habitual drives are largely automatic for the expert driver, habitual self-control challenges will also be largely automatic for the skillful self-controlled agent, who will control performance at an abstract level and allow well-tuned automated routines to do most of the work. That said, even experts eventually have to face extraordinary circumstances (e.g. personal crises, extreme turns of luck) that test their abilities, and in those cases they will need to exert much closer agentive control over the situation.

One of the instances where flexible practical reasoning is necessary is when agents must switch strategies. This is an under-explored but common feature of self-control exertions admitting of individual variation (Ford et al. 2019). During marshmallow studies, children attempt strategy switches to varying degrees, and those who make more distinct attempts and try more diverse strategies tend to have greater success at delaying gratification (Carlson and Beck 2008; Haimovitz et al. 2019). The job of agentive guidance thus continues while self-control exertion lasts, and often requires attempting multiple strategies in a single episode.

### Supporting evidence

If what I have said so far is right, the skill of self-control involves correctly forming and managing mixed-format intentions via flexible practical reasoning. This provides a crucial test for the account: if the abilities involved in flexible practical reasoning are characteristic of self-controlled agents, then differences in how agents form and manage intentions should make a difference in their success at self-control. For example, some people might form more detailed intentions than others; and some people might differently represent the motivational states justifying their intentions. If the skill model proposed above is correct, such differences should be correlated with differences in self-control success. I will briefly discuss two lines of empirical research that corroborate this point.

A quite direct corroboration comes from research on *implementation intentions:* commitments to satisfy a general intention by carrying out a specific action plan in a specific situation (Gollwitzer 1999). An implementation intention contains an if–then plan that specifies “when, where, and how the person will instigate responses that promote goal realization” (Gollwitzer and Sheeran 2006). Large bodies of work have consistently shown that explicitly forming implementation intentions positively affects goal attainment by strengthening both action initiation and perseverance in goal pursuit in the face of temptations (Ib.). Recent meta-analyses corroborate a robust, positive effect of implementation-intention formation on overcoming temptations in realms like increasing physical activity (Bélanger-Gravel et al. 2013), quitting smoking (McWilliams et al. 2019), and adopting healthier diets (Vilà et al. 2017).

Thus, agents who purposefully form a detailed mixed-format intention (a specific set of instructions about the steps of strategy implementation) can structure lower-level routines more robustly and effectively. Arguably this occurs because automatic control processes can monitor the implementation of detailed intentions more closely, and can thus produce clearer and more numerous action-oriented feelings that the agent is then able to use for guiding performance through flexible practical reasoning.

Another source of support can be found in research on motivation framing, which shows that when pursuing a goal, it matters not only how motivated you are, but also how you represent the motivations underlying your intention. Agents can be chiefly motivated to pursue a goal or an action by either *want-to motivations* (by considering the goal or action self-rewarding, inherently enjoyable or linked to their core values or identity) or *have-to motivations* (by linking the goal or action to extrinsic incentives like monetary pay, the avoidance of punishment or of negative emotions like guilt or shame) (Werner and Milyavskaya 2019). Participants motivated by want-to motivations report lower levels of temptation to quit, and are willing to persevere in the tasks for longer periods (Koestner et al. 2002; Milyavskaya et al. 2015). Moreover, people who construe their goals as propelled by want-to motivations tend to have higher trait self-control scores (Converse et al. 2019).

Werner and Milyavskaya (2019) suggest motivation framing affects performance by moderating feelings of effort: “want-to goals feel subjectively easier, even when the goal requires the same amount of objective effort”. Thus it seems agents who frame a self-control exertion as motivated by their own tastes and values (instead of framing it as an instrumental means for a distinct end) are able to persevere in its production for longer periods and can dedicate more cognitive resources to its performance. So there is initial evidence that the way people frame their motivations affects the way strategy selectors calculate the expected costs and benefits of action. This is reflected in the agents’ experiencing fewer temptations and lower FME levels under a want-to framing than under a have-to framing. Skillful practical reasoners would be able to use such framing effects to their advantage, selecting the framing that allows them to implement control strategies most effectively.

This allows for an expansion of an already-existing idea. Holton (2009, p. 123) claims that agents must rehearse their intentions in order to overcome temptation. Rehearsal involves reminding oneself of one’s intention and the reasons for having formed it—thereby maintaining their influence on performance, without re-opening deliberation—since the latter would undermine the intention’s role as a commitment. Motivation-framing research suggests that skilled agents can do much more than rehearse: they can *reframe* their intentions and reasons, i.e. search for alternative descriptions of their relevant mental states looking for the ones that best trigger and sustain the intended performance. Switching motivation from a have-to to a want-to framing would be a specific reframing technique.

It could be objected that even if want-to goal framing is associated with self-control success, it does not show that agents intentionally engage in reframing; some might simply be more disposed to want-to framing than others. The evidence mentioned above does not in fact show that agents intentionally engage in flexible practical reasoning to alter the framing of their motivational states. However, research on the strategy of cognitive reappraisal systematically shows that one can become better at self-control by learning to reframe diverse aspects of one’s situation—including one’s motivations (Gross 2015, p. 9).

Evidence thus corroborates the claim that the ability to appropriately form and shape mixed-format intentions allows agents to exert self-control more effectively. This shows that the skill of self-control involves the ability to manage intentions via flexible practical reasoning.

## The skill model of self-control

This section specifies ways in which the skill model furthers our understanding of self-control. I highlight two upshots of the skill model: (4.1) the primacy of synchronic self-control and (4.2) the prevalence of diachronic self-control.

### The primacy of synchronic self-control

Mylopoulos and Pacherie [M&P] (2020) characterize self-control as a “hybrid skill”. The skill model developed here shares general commitments with M&P’s approach: both accounts portray self-control as a hybrid skill (one which merges top-down, reflective processes and spontaneous, automatic processes), and both explain self-control exertion by specifying the multiple levels of control that make it possible. There are, however, crucial differences. M&P’s focus is on diachronic self-control (the ability to solve foreseeable motivational conflicts), whereas I focus here on synchronic self-control (the ability to solve current motivational conflicts).^19^ Since M&P focus on diachronic self-control, they concentrate their discussion of control types on *strategic control:* “the ability to anticipate a potential loss of control given the upcoming situation of action” (p.93). They argue convincingly that this kind of control depends crucially on (1) the agent’s knowledge of her own strengths and weaknesses, necessary to anticipate motivational conflicts; and (2) her tendency to think about the future.

Following this line of thought, I take the skill model to explain how condition (1) can be fulfilled. Diachronic self-control must depend on the processes whereby the agent acquires regulatory self-knowledge, and the monitoring mechanisms of synchronic self-control are a reliable source of knowledge of precisely that kind. Trained to detect conflicts and assess the expected costs and benefits of regulating them, these mechanisms also learn to associate specific situation types with expected regulatory risks, in a way that can be deployed preventively upon encountering the situation in the future. To go back to Tom, after struggling with wine-related temptations a few times while getting groceries, he may begin to anticipate a potential loss of control *before* entering the store. This would allow him to exercise some strategic control of the M&P variety: he might intentionally avoid the wines section, or preventively promise Tim he will not buy any wine this time.

If this is correct, then the self-knowledge required to anticipate a potential temptation emerges from the accumulation of information by the automatic monitoring mechanisms underlying synchronic self-control exertions. Just as they output personal-level feelings of effort that predict the costs and benefits of control exertion, these mechanisms can also output action-oriented feelings of alertness or risk, emotional warnings that motivation conflicts might arise in a given situation. If the agent is sufficiently prone to future-oriented thinking (M&P’s condition (2)), these feelings will allow her to devise strategies to tackle the motivational conflict before it becomes too strong.

We can then say that synchronic self-control is explanatorily prior to diachronic self-control. This is because it will be possible for agents to begin anticipating the regulatory demands of familiar situations once they have acquired the monitoring capacities constitutive of synchronic self-control. Such capacities make it possible to anticipate motivational conflicts, thereby making diachronic self-control possible.

This suggests that action-oriented representations play two key roles in self-regulation: first, by representing the expected costs and benefits of control strategy implementation, they have the proximal function of enabling the skilled guidance of synchronic self-control exertions via flexible practical reasoning. And by representing the accumulated self-knowledge about the agent’s own motivational strengths and weaknesses, and about which kinds of situations are likely to exploit the latter, they also have the distal function of making anticipatory self-regulation strategies possible. Proximally, action-oriented representations contribute to the exercise of synchronic self-control (the skill of overcoming currently-felt strong temptations), while distally they contribute to the development and exercise of diachronic self-control (the skill of minimizing or avoiding foreseeable temptations).

### The prevalence of diachronic self-control

If the self-knowledge constituted by the accumulated practice of synchronic self-control exertions enables the development of diachronic regulatory strategies, then skilled self-controlled agents will have better chances to devise and deploy anticipatory strategies that seek to minimize or altogether avoid temptations. There is reason to expect diachronic strategies to be more efficient in the long run, since they minimize the effort of intense cognitive control usage (Duckworth et al. 2016): developing steady and strong exercise habits is less resource-consuming than effortfully dragging oneself to the gym every time one wants to work out. It is therefore likely that agents skilled at diachronic self-control will tend to select preventive, diachronic strategies over reactive, synchronic ones.

This ultimately leads to a somewhat surprising result: agents more skilled at synchronic self-control will tend to have more diachronic tools at their disposal; and so, since these tend to be less effortful, they will tend to select diachronic strategies more often than synchronic ones. Synchronic strategies will perhaps be necessary for unexpected or unusually strong motivational conflicts, but frequent motivational conflicts will tend to be avoided or minimized pre-emptively rather than allowed freely to initiate and then faced head-on.

Thus, the skill model suggests the prevalence of diachronic self-control strategies in skilled agents. This is in fact supported by evidence suggesting that long-term goal attainment is correlated, not with more successfully resisting temptation, but with feeling tempted less frequently (Hofmann et al. 2012; Milyavskaya and Inzlicht 2017). Additionally, trait self-control measures are robustly correlated with the tendency to engage in planning (Sjåstad and Baumeister 2018). It thus seems that those who excel at exerting self-control tend to rely on diachronic strategies more frequently than on synchronic ones. This suggests that synchronic self-control is a sort of self-effacing skill: the more skilled you are at it, the less often you tend to use it. Its crucial long-term role seems to be to enable diachronic regulation by providing the self-knowledge required to anticipate motivational conflicts. Beyond that, for skilled agents synchronic strategies would be an emergency measure rather than an everyday feature of self-regulation.

To further strengthen the claim that synchronic self-control is self-effacing, consider the relationships between self-control and habit. People who with high trait self-control measures seem at the same time to have weaker habits for unhealthy activities (Adriaanse et al. 2014), and to be able to develop more robust commitment-consistent habits (Galla and Duckworth 2015). This further supports the view that skilled agents, instead of relying on their advanced synchronic strategies, tend instead to use diachronic ones (like planning and situation selection) to form and manage habits, which, once established, largely automate self-regulation and minimize the need for synchronic self-control (Carden and Wood 2018).

Thus, although synchronic self-control is explanatorily prior, diachronic self-control is prevalent in practice for skilled agents. This highlights a remarkable contrast between self-control and other forms of skill: the more skilled an agent is at synchronic self-control, the less she will tend to use the synchronic strategies that constitute that skill. This is unlike standard cases of motor skills (like driving or playing basketball), in which the agent continuously refines and structures action representations, motor schemas, and internal models of the same action domain as she improves her skill. In the case of self-control, when agents have sufficiently developed their synchronic skill, they tend to replace it with a diachronic one.^20^

It makes sense that this is so. After all, self-control is arguably a purely instrumental skill: we use it merely to disable temptations that threaten to undermine the consistency between our commitments and our actions, so we exert self-control only to the extent that we need to avoid motivational obstacles. Thus, if there are no motivational obstacles, there is no need for self-control. In fact, the less self-control we need to exert the better, since minimizing it would liberate cognitive resources to pursue the commitments and goals that we value non-instrumentally. So, while it is better to exert synchronic self-control than to give in to an active temptation, it is better still to exert diachronic self-control than to exert synchronic self-control (insofar as the former is more resource-efficient than the latter); and even better to be able to rely on habits for self-regulation (for the same reason). It is therefore rational for synchronic self-control to be self-effacing: doing so frees us to focus on what we actually care about.

## Conclusion

This paper began with the challenge of constructing an account of self-control capable of fulfilling the skillful agency requirement and tackling the guidance problem. The resulting skill model holds that self-control involves the ability to engage in flexible practical reasoning to manage mixed-format intentions as the self-control exertion unfolds. Evidence of the effects of implementation intentions and motivation framing on self-control success corroborates that mastering our temptations requires mastering the flexible practical deliberation that structures and threads together cognitive-control processes as they unfold.

The skill model developed here fills a key gap in self-control research. Many agree that self-control exertions are skilled actions, but no account of such skilled agency was previously available, particularly for cases of synchronic self-control. The skill model specifies how such skilled action unfolds. I have additionally shown that, while synchronic self-control has developmental primacy, diachronic forms of regulation have more prevalence as skill increases, and that self-control is thus a self-effacing skill. This helps to harmonize the apparently conflicting claims that self-control exertions depend on cognitive control, and that those who succeed most at exerting self-control rely more on automaticity and habit than on cognitive control.

## Data Availability

This is a theoretical paper; no novel data or materials were gathered.

## Footnotes

1 Recent notable exemplars include K. McGonigal’s *The willpower instinct* (Penguin, 2011); B. Tracy’s *No excuses!: The power of self-discipline* (2010, Vanguard); A. Johnson’s *The little book of big change: The no-willpower approach to breaking any habit* (2016, New Harbinger); and R. Baumeister and J. Tierney’s *Willpower: Rediscovering the greatest human strength* (Penguin, 2012).

2 Mylopoulos and Pacherie (2020) take a notable step in this direction, albeit their focus (diachronic self-control) is different from this work’s (synchronic self-control). For discussion see Sect. 4.1 below.

3 These  are usually understood to be ‘proximal’ temptations and commitments, i.e. they are about what the agent is to do now (as opposed to some other future time) (Kennett and Smith 1996; Mele 1987, ch. 5, 2003 ch. 8; Sripada 2014).

4 This leads back at least to Aristotle (*Eudemian Ethics* II). One could reply that we also sometimes praise and blame people for things that are not actions, like desires or character traits. However, arguably the non-actional things we hold ourselves responsible for are to some extent under our control, were originated by things that were under our control, or are displays of things under our control.

5 In saying this I am not committed to the stronger claim that all instances of self-control exertion are instances of skillful action. E.g. self-control could sometimes be a matter of “brute force” resistance (Mele 1987).

6 Self-control may also be said to tackle commitment-discordant habits (Silver 2019). I leave that possibility aside here and focus solely on self-control in commitment/temptation conflicts.

7 Arguably a part of the Western-philosophical discussion since Plato and Aristotle’s divisions between the soul’s passionate and rational elements in the *Republic* and the *Nicomachean Ethics*, divided-mind accounts have recently enjoyed a notorious resurgence inspired by dual-process models in cognitive science (Evans and Stanovich, 2013). Contemporary versions include Haas (2018), Heatherton and Wagner (2011); Hofmann et al. (2009); Holton (2009), Levy (2011, 2017) and Sripada (2014). For critical discussion see Mele (2014). While a connection between cognitive control and self-control is often simply assumed (Inzlicht et al. 2020), Sripada (2020) develops a detailed theory that bridges the two concepts.

8 This is a simplification of Sripada’s view, according to which cognitive control can tackle only millisecond-fast “response pulses”, whereas temptations are identifiable with the more persistent “emotion-type states” that originate such pulses. Only response pulses can be directly controlled, however, so exerting self-control over the temptation requires skillfully deploying cognitive control against the myriad response pulses it produces.

9 Sequential-sampling models are also used by others to account for self-control (Ainslie 2020; Berkman et al. 2017). There too the decision-making process remains subpersonal.

10 This is the so-called “interface problem” (Butterfill and Sinigaglia 2014). (For discussion see Brozzo 2017; Fridland 2019; Mylopoulos and Pacherie 2017; Shepherd 2017.) The interface problem and the guidance problem share a common general concern: defining how explicit and implicit processes work together to coordinate action. If it is agreed that commitments are propositionally formatted while the processes constituting EVC calculations have a non-propositional, reinforcement-based associative format, then the guidance problem in self-control would constitute a version of the interface problem.

11 To be clear, nothing here hinges on this specific framework being correct for motor action. I introduce it because its conceptual structure will be helpful to solving the guidance problem for self-control in §3.

12 For other models see Butterfill and Sinigaglia (2014) and Mylopoulos and Pacherie (2017). I leave the debates surrounding these proposals aside (referring the interested reader to the review in Mylopoulos and Pacherie 2019) and focus on mixed-formats models because they rely on the claim that motor representations, although non-propositional in format, are however conceptual in content, and are thus subject to rationality constraints and capable of entering into practical reasoning processes. This will be useful when returning to self-control.

13 The distinction between general and mixed-format intentions closely follows Fridland’s (2019) general-intention / practical-intention distinction. Notice this is not a temporal distinction (unlike e.g. Bratman’s (1987) future-directed vs. present-directed intentions): general and mixed-format intentions coexist during action performance, simultaneously structuring it at different levels.

14 Flexible practical reasoning can also take a mixed-format intention as starting point, and seek to form a more specific mixed-format intention. For the sake of simplicity, I focus on the case of general intentions as starting points.

15 This example and Fig. 1 are adapted from Christensen et al. (2016).

16 These four levels can be subdivided into multiple sub-levels within the four categories.

17 I thank an anonymous reviewer for alerting me to the need to tackle these questions.

18 This way of framing flexible practical reasoning borrows from Koralus’ (2014) erotetic theory of attention. On the link between skillful action and answering ‘How to?’ questions, see Douskos (2019).

19 Some researchers deny that diachronic strategies should be considered forms of self-control (Sripada 2020). I have no space here to address this point. Suffice it to say that nothing here hinges on whether diachronic regulation is a form of self-control. If preferred, replace ‘diachronic self-control’ with ‘diachronic self-regulation’.

20 I thank an anonymous reviewer for pressing me to clarify the contrast between self-control and other skills.

## References

[CR1] Adriaanse MA, Kroese FM, Gillebaart M, De Ridder DTD (2014). Effortless inhibition: Habit mediates the relation between self-control and unhealthy snack consumption. Frontiers in Psychology.

[CR2] Ainslie, G. (2020). Willpower with and without effort. *Behavioral and Brain Sciences*. 10.1017/S0140525X20000357 PMC9280284 32843105

[CR3] Aldao A, Nolen-Hoeksema S (2013). One versus many: Capturing the use of multiple emotion regulation strategies in response to an emotion-eliciting stimulus. Cognition and Emotion.

[CR4] Aldao A, Nolen-Hoeksema S, Schweizer S (2010). Emotion-regulation strategies across psychopathology: A meta-analytic review. Clinical Psychology Review.

[CR5] Bélanger-Gravel A, Godin G, Amireault S (2013). A meta-analytic review of the effect of implementation intentions on physical activity. Health Psychology Review.

[CR72] Bermúdez, J. P. (2021). Willpower needs tactical skill.* Behavioral and Brain Sciences*. 10.1017/S0140525X20000898. 10.1017/S0140525X20000898 33899708

[CR6] Berkman ET, Hutcherson CA, Livingston JL, Kahn LE, Inzlicht M (2017). Self-control as value-based choice. Current Directions in Psychological Science.

[CR7] Bonanno GA, Burton CL (2013). Regulatory flexibility: An individual differences perspective on coping and emotion regulation. Perspectives on Psychological Science.

[CR8] Bratman M (1987). Intention, plans, and practical reason.

[CR9] Briscoe RE, MacPherson F, Dorsch F (2018). Superimposed mental imagery: On the uses of make-perceive. Perceptual imagination and perceptual memory.

[CR10] Brozzo C (2017). Motor intentions: How intentions and motor representations come together. Mind & Language.

[CR11] Butterfill SA, Sinigaglia C (2014). Intention and motor representation in purposive action. Philosophy and Phenomenological Research.

[CR12] Carden L, Wood W (2018). Habit formation and change. Current Opinion in Behavioral Sciences.

[CR13] Carlson, S. M., & Beck, D. M. (2008). Symbols as tools in the development of executive function. *23*, 409–417.

[CR14] Christensen W, Sutton J, Mcilwain DJF (2016). Cognition in skilled action: Meshed control and the varieties of skill experience. Mind and Language.

[CR15] Converse BA, Juarez L, Hennecke M (2019). Self-control and the reasons behind our goals. Journal of Personality and Social Psychology.

[CR16] Douskos C (2019). The spontaneousness of skill and the impulsivity of habit. Synthese.

[CR17] Duckworth AL, Gendler TS, Gross JJ (2016). Situational strategies for self-control. Perspectives on Psychological Science.

[CR18] Evans JSBT, Stanovich KE (2013). Dual-process theories of higher cognition: Advancing the debate. Perspectives on Psychological Science.

[CR19] Ford BQ, Gross JJ, Gruber J (2019). Broadening our field of view: The role of emotion polyregulation. Emotion Review.

[CR20] Forstmann BU, Ratcliff R, Wagenmakers E-J (2016). Sequential sampling models in cognitive neuroscience: Advantages, applications, and extensions. Annual Review of Psychology.

[CR21] Frankfurt HG (1978). The problem of action. American Philosophical Quarterly.

[CR22] Fridland, E. (2019). Intention at the interface. *Review of Philosophy and Psychology*.

[CR23] Galla BM, Duckworth AL (2015). More than resisting temptation: Beneficial habits mediate the relationship between self-control and positive life outcomes. Journal of Personality and Social Psychology.

[CR24] Gollwitzer PM (1999). Implementation intentions: Strong effects of simple plans. American Psychologist.

[CR25] Gollwitzer PM, Sheeran P (2006). Implementation intentions and goal achievement: A meta-analysis of effects and processes. Advances in Experimental Social Psychology.

[CR26] Gross JJ (2015). Emotion regulation: Current status and future prospects. Psychological Inquiry.

[CR27] Haas J (2018). An empirical solution to the puzzle of weakness of will. Synthese.

[CR28] Haimovitz, K., Dweck, C. S., & Walton, G. M. (2019). Preschoolers find ways to resist temptation after learning that willpower can be energizing. *Developmental Science*. 10.1111/desc.12905 31529554

[CR29] Heatherton TF, Wagner DD (2011). Cognitive neuroscience of self-regulation failure. Trends in Cognitive Sciences.

[CR30] Hofmann W, Baumeister RF, Förster G, Vohs KD (2012). Everyday temptations: An experience sampling study of desire, conflict, and self-control. Journal of Personality and Social Psychology.

[CR31] Hofmann W, Friese M, Strack F (2009). Impulse and self-control from a dual-systems perspective. Perspectives on Psychological Science.

[CR32] Holton R (2009). Willing, wanting, waiting.

[CR33] Inzlicht, M., Werner, K. M., Briskin, J. L., & Roberts, B. W. (2020). Integrating models of self-regulation. *Annual Review of Psychology*. 10.1146/annurev-psych-061020-105721 33017559

[CR34] Kennett J, Smith M (1996). Frog and Toad lose control. Analysis.

[CR35] Koestner R, Lekes N, Powers TA, Chicoine E (2002). Attaining personal goals: Self-concordance plus implementation intentions equals success. Journal of Personality and Social Psychology.

[CR36] Kool W, McGuire JT, Rosen ZB, Botvinick MM (2010). Decision making and the avoidance of cognitive demand. Journal of Experimental Psychology: General.

[CR37] Koralus P (2014). The erotetic theory of attention: Questions, focus and distraction. Mind and Language.

[CR38] Kurzban R, Duckworth A, Kable JW, Myers J (2013). An opportunity cost model of subjective effort and task performance. Behavioral and Brain Sciences.

[CR39] Levy N (2011). Resisting “weakness of the will”. Philosophy and Phenomenological Research.

[CR40] Levy N, Sinnott-Armstrong W, Miller CB (2017). Of marshmallows and moderation. Moral psychology: Virtue and character.

[CR41] McWilliams L, Bellhouse S, Yorke J, Lloyd K, Armitage CJ (2019). Beyond “planning”: A meta-analysis of implementation intentions to support smoking cessation. Health Psychology.

[CR42] Mele AR (1987). Irrationality: An essay on akrasia, self-deception, and self-control.

[CR43] Mele AR (1998). Underestimating self-control: Kennett and Smith on Frog and Toad. Analysis.

[CR44] Mele AR (2003). Motivation and agency.

[CR45] Mele AR, Gallagher S (2011). Self-control in action. Oxford handbook of the self.

[CR46] Mele AR (2014). Self-control, motivational strength, and exposure therapy. Philosophical Studies.

[CR47] Metcalfe J, Mischel W (1999). A hot/cool-system analysis of delay of gratification: Dynamics of willpower. Psychological Review.

[CR48] Milyavskaya M, Inzlicht M (2017). What’s so great about self-control? Examining the importance of effortful self-control and temptation in predicting real-life depletion and goal attainment. Social Psychological and Personality Science.

[CR49] Milyavskaya M, Inzlicht M, Hope N, Koestner R (2015). Saying “no” to temptation: Want-to motivation improves self-regulation by reducing temptation rather than by increasing self-control. Journal of Personality and Social Psychology.

[CR50] Mischel W, Gollwitzer PM, Bargh JA (1996). From good intentions to willpower. The psychology of action.

[CR51] Mischel W (2014). The marshmallow test: Understanding self-control and how to master it.

[CR52] Mischel W, Shoda Y, Rodriguez M (1989). Delay of gratification in children. Science.

[CR53] Moffitt TE, Arseneault L, Belsky D, Dickson N, Hancox RJ, Harrington H, Houts R, Poulton R, Roberts BW, Ross S, Sears MR, Thomson WM, Caspi A (2011). A gradient of childhood self-control predicts health, wealth, and public safety. Proceedings of the National Academy of Sciences.

[CR54] Mylopoulos M, Pacherie E (2017). Intentions and motor representations: The interface challenge. Review of Philosophy and Psychology.

[CR55] Mylopoulos, M., & Pacherie, E. (2019). Intentions: The dynamic hierarchical model revisited. *Wiley Interdisciplinary Reviews: Cognitive Science*, *10*(2). 10.1002/wcs.1481 30105894

[CR56] Mylopoulos M, Pacherie E, Mele AR (2020). Self-control as hybrid skill. Surrounding self-control.

[CR57] Naragon-Gainey K, McMahon TP, Chacko TP (2017). The structure of common emotion regulation strategies: A meta-analytic examination. Psychological Bulletin.

[CR58] Pacherie E (2008). The phenomenology of action: A conceptual framework. Cognition.

[CR59] Pacherie, E., & Mylopoulos, M. (2020). Beyond automaticity: The psychological complexity of skill. *Topoi*.

[CR60] Roskies AL (2018). Decision-making and self-governing systems. Neuroethics.

[CR61] Shenhav A, Botvinick MM, Cohen JD (2013). The Expected Value of Control: An integrative theory of anterior cingulate cortex function. Neuron.

[CR62] Shepherd J (2017). Skilled action and the double life of intention. Philosophy and Phenomenological Research.

[CR63] Shepherd, J. (2018). Intelligent action guidance and the use of mixed representational formats. *Synthese*. 10.1007/s11229-018-1892-7 PMC8550228 34720227

[CR64] Silver K (2019). Habitual weakness. Thought: A Journal of Philosophy.

[CR65] Sjåstad H, Baumeister RF (2018). The Future and the Will: Planning requires self-control, and ego depletion leads to planning aversion. Journal of Experimental Social Psychology.

[CR66] Sripada C (2014). How is willpower possible? The puzzle of synchronic self-control and the divided mind. Nous.

[CR67] Sripada, C. (2020). The atoms of self-control. *Noûs*.

[CR68] Tabibnia G, Monterosso JR, Baicy K, Aron AR, Poldrack RA, Chakrapani S, Lee B, London ED (2011). Different forms of self-control share a neurocognitive substrate. Journal of Neuroscience.

[CR69] Vilà I, Carrero I, Redondo R (2017). Reducing fat intake using implementation intentions: A meta-analytic review. British Journal of Health Psychology.

[CR70] Werner KM, Milyavskaya M (2019). Motivation and self-regulation: The role of want-to motivation in the processes underlying self-regulation and self-control. Social and Personality Psychology Compass.

[CR71] Westbrook A, Braver TS (2015). Cognitive effort: A neuroeconomic approach. Cognitive, Affective and Behavioral Neuroscience.

